# PPARs and the Kynurenine Pathway in Melanoma—Potential Biological Interactions

**DOI:** 10.3390/ijms24043114

**Published:** 2023-02-04

**Authors:** Katarzyna Walczak, Agnieszka Gerkowicz, Dorota Krasowska

**Affiliations:** 1 Laboratory for Immunology of Skin Diseases, Chair and Department of Dermatology, Venerology and Paediatric Dermatology, Medical University of Lublin, Radziwillowska 11, 20-080 Lublin, Poland; 2 Chair and Department of Dermatology, Venerology and Paediatric Dermatology, Medical University of Lublin, Staszica 11Ł, 20-081 Lublin, Poland; agnieszka.gerkowicz@umlub.pl (A.G.); dor.krasowska@gmail.com (D.K.)

**Keywords:** peroxisome proliferator-activated receptor, melanoma, kynurenine pathway, kynurenine, kynurenic acid, proliferation, cell death, cell cycle, metabolism

## Abstract

Peroxisome proliferator-activated receptors (PPARs) are ligand-activated transcription factors involved in various physiological and pathological processes within the skin. PPARs regulate several processes in one of the most aggressive skin cancers, melanoma, including proliferation, cell cycle, metabolic homeostasis, cell death, and metastasis. In this review, we focused not only on the biological activity of PPAR isoforms in melanoma initiation, progression, and metastasis but also on potential biological interactions between the PPAR signaling and the kynurenine pathways. The kynurenine pathway is a major pathway of tryptophan metabolism leading to nicotinamide adenine dinucleotide (NAD+) production. Importantly, various tryptophan metabolites exert biological activity toward cancer cells, including melanoma. Previous studies confirmed the functional relationship between PPAR and the kynurenine pathway in skeletal muscles. Despite the fact this interaction has not been reported in melanoma to date, some bioinformatics data and biological activity of PPAR ligands and tryptophan metabolites may suggest a potential involvement of these metabolic and signaling pathways in melanoma initiation, progression, and metastasis. Importantly, the possible relationship between the PPAR signaling pathway and the kynurenine pathway may relate not only to the direct biological effect on melanoma cells but also to the tumor microenvironment and the immune system.

## 1. Introduction

Peroxisome proliferator-activated receptors (PPARs) are ligand-activated transcription factors. Together with receptors for steroids, vitamin D, retinoid X receptor, and thyroid hormone, PPARs belong to the family of nuclear hormone receptors [1,2]. So far, three isoforms of PPARs have been identified: PPARα, PPARγ, and PPARβ/δ. Each of them is encoded by different genes [3] and demonstrates diversity in ligand specificities, tissue distribution, and biological role [4,5,6]. PPARα is detected in metabolically active tissues, such as the liver, heart, brown adipose tissue, skeletal muscle, kidneys, and intestinal mucosa. This receptor is involved in β-oxidation of fatty acids; its activation leads to a decrease in lipid level [1,4,7]. *PPARG* gene, coding PPARƴ, is expressed in white and brown adipose tissue, liver, spleen, heart, sebaceous glands, pancreas, prostate, retina, keratinocytes, dendritic cells, activated macrophages, and lymphocytes [1,2,4]. PPARƴ contributes to lipid storage, maturation, and differentiation of adipocytes and glucose homeostasis. Importantly, this isoform has a regulatory effect on inflammatory processes [1,2,4]. PPARγ occurs in two isoforms: PPARƴ1 and PPARƴ2 [1,2,4,8]. PPARƴ1 is expressed in most cells, whereas expression of PPARƴ2 is limited to adipocytes [9]. Importantly, PPARƴ2 is a more potent transcription factor than the second isoform. Interestingly, previous studies identified three messenger RNA (mRNA) of *PPARG* gene due to its separate promoters and 5′ exons [10]. However, *PPARƴ1* and *PPARƴ3* encode the same protein, PPARƴ1. *PPARD* gene, coding PPARβ/δ, is expressed mainly in the liver, gastrointestinal tract, kidneys, skin, abdominal adipose tissue, and skeletal muscles. This receptor is responsible for glucose homeostasis, proliferation and differentiation of adipocytes, and lipid metabolism in the brain [1,2,4].

## 2. PPARs in the Skin

**Figure 1:**
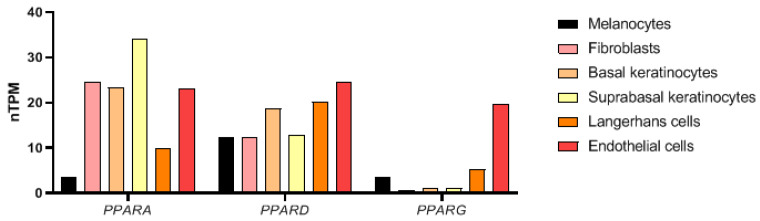
RNA expression of *PPARA*, *PPARD*, and *PPARG* in the single type clusters based on the Human Protein Atlas [15]. The data show gene expression in different cell types within the skin representing the following clusters: melanocytes c-13, fibroblasts c-1, basal keratinocytes c-5, suprabasal keratinocytes c-6, Langerhans cells c-0, endothelial cells c-4. The results are presented as normalized transcript expression values (nTPM—normalized transcripts per million), calculated for each gene in every sample (values <0.1 are not visualized). All information concerning the bioinformatics analysis of clustering of single cell transcriptomic data and single cell transcriptomic datasets is available at the Human Protein Atlas website (https://www.proteinatlas.org/about/assays+annotation (accessed on 17 January 2023)).

PPARs are essential in various physiological and pathological processes within the skin. They are involved not only in skin metabolic homeostasis but also in melanogenesis, cell proliferation, differentiation, apoptosis, immune response, and inflammation, presenting both pro- and anti-inflammatory activity within the skin and pilosebaceous unit. Genetic analysis indicated the expression of *PPARA*, *PPARD*, and *PPARG* genes in various skin cells (Figure 1); however, previous studies revealed that gene expression is not directly correlated to the protein level of these receptors [11]. All three PPAR isoforms were found in the epidermis, among which PPARβ/δ is a prevalent subtype. Nevertheless, increased expression of all PPARs’ isoforms was reported during the differentiation of keratinocytes [1]. Mao-Qiang et al. [12] demonstrated that topical application of PPARy activators, ciglitazone and troglitazone, to mouse skin stimulated epidermal differentiation. In accordance with in vivo study, ciglitazone increased the expression of genes coding involucrin and transglutaminase 1, markers of differentiations, in human keratinocytes [12]. Increased expression of keratinocyte differentiation markers was also observed after exposure to non-selective PPARβ/δ agonist and PPARβ/δ-selective ligand [2,13]. Activation of PPARα and PPARβ/δ has been reported to play a crucial role in skin barrier function by regulating differentiation and lipid synthesis in keratinocytes [14].

Previous studies revealed that PPARs are also involved in melanocyte proliferation and melanogenesis. Kang et al. [16] reported that activation of PPARƴ and PPARα by ciglitazone and WY-14643, respectively, inhibited the proliferation of melanocytes in a dose-depended manner but simultaneously increased melanin biosynthesis. Additionally, Lee et al. [17] confirmed increased pigmentation after the administration of PPARƴ agonist, ciglitazone, in cultured human melanocytes and cultured skin. Moreover, ciglitazone enhanced the migration of human melanocytes [17].

On the other hand, the possible involvement of PPARs was implicated in the pathogenesis of psoriasis, atopic dermatitis, acne vulgaris, lichen planopilaris, actinic keratosis, and skin cancers including squamous cell carcinoma and melanoma [1,2,4,18]. However, this review will be focused on the biological role of PPARs in melanoma.

## 3. PPARs and Melanoma

Melanoma (malignant melanoma) is one of the most aggressive skin cancer, characterized by increasing incidence and high mortality in humans, which makes melanoma the 15th most common cancer in the world [19,20,21]. Surgical resection of melanoma in its early stages is associated with a 90% 5-year survival rate. However, advanced melanoma tends to metastasize more likely than other skin cancers, with the lung being the most common localization for distant metastases [22]. The presence of metastases is a negative factor for overall treatment outcome and survival rate [20,21].

Eastham et al. reported that all *PPAR* genes were expressed in melanocytes and melanoma cells [11]. Notably, the protein levels of PPARα and PPARγ were higher in mouse and human melanoma cells than in normal melanocytes [11]. Interestingly, there was no significant correlation between gene expression and protein level of PPARα. Additionally, the differences in the protein level of PPARβ in melanocytes and various types of melanoma were demonstrated. PPARβ was localized in the nucleus in melanocytes, whereas its localization pattern in melanoma samples was more heterogeneous [23]. Moreover, the protein level of PPARβ was lower in superficial spreading melanomas than in nodular melanomas and melanoma metastasis [23].

PPARs are involved in various processes in melanoma, including melanogenesis [16,24]. Melanoma pigmentation and fully functional melanogenic apparatus are significantly associated with increased resistance to radio- and chemotherapy [25]. The protein expression of PPARs was also investigated in melanoma. Mössner et al. confirmed the presence of PPARƴ in primary melanoma, its metastases, and human melanoma cell lines, including MM-201, MM-254, KAII, and MM-358 [26]. Moreover, Grabacka et al. demonstrated a negative correlation between PPARα and melanin synthesis in murine melanoma cells B16F10 [25].

PPARs are also involved in cell cycle regulation and proliferation of melanoma cells. The majority of studies are focused on PPARγ activity towards melanoma cells. Agonists of PPARγ, including ciglitazone, troglitazone, rosiglitazone, pioglitazone and 15d-PGJ2 inhibited proliferation of melanoma cell lines representing different stages of cancer progression [11,26,27,28]. The crucial role of PPARγ in the proliferation of melanoma cells has been previously reported by Smith et al. [29]. Disruption in the PPARγ signaling pathway by siRNA led to attenuation of antiproliferative activity of thiazolidinediones: troglitazone and halofenate [29]. Importantly, the antiproliferative potential of PPARγ agonists has also been observed in vivo [30,31,32]. Dana et al. reported that PPARƴ agonist pioglitazone significantly reduced melanoma cell proliferation and tumor size in mice [33]. Additionally, treatment of nude mice with ciglitazone dramatically inhibited human melanoma xenograft development [30]. Previous studies revealed that the anticancer potential of PPARγ ligands resulted from the negative regulation of the cell cycle [34].

The antiproliferative activity of PPAR ligands resulted from various molecular mechanisms. It was suggested that activation of PPARƴ inhibited the proliferation of melanoma cells and induced apoptosis via inhibiting the Toll-like receptor-4 (TLR-4)-dependent NF-κB pathway [33]. Paulitschke et al. supported previous reports of PPARγ agonists describing both a direct anti-tumor and a broad spectrum of anti-stromal, anti-angiogenetic, and immuno-modulating activities [35]. On the contrary, Meylan et al. demonstrated that PPARγ activation by rosiglitazone might be associated with carcinogenesis [36]. Activation of PPARγ led to reduced expression of thioredoxin-interacting protein (*TXNIP*) in human melanoma A375 cells. Similarly, patients with primary or metastatic melanoma had significantly lower expression of *TXNIP* within the lesion compared to benign melanocytic naevi and healthy control. Thus, it was suggested that reduced *TXNIP* expression is associated with melanoma progression and exerts a pro-tumorigenic effect [36].

The role of PPARβ/δ in carcinogenesis remains inconclusive since previous studies confirmed both pro-tumorigenic and anti-tumorigenic effects of PPARβ/δ activation [22,23,37]. Activation of PPARβ/δ by either GW501516 or GW0742 was demonstrated to inhibit the proliferation of human melanoma UACC903 and A375 cells as well as mouse melanoma B16F0 cells [23,37]. Additionally, Lim et al. reported that inhibition of PPARβ/δ signaling by 10h antagonist led to the transformation of B16F10 melanoma cells from typical shape to elongated mesenchymal-like structure, which is characteristic for invasive melanoma cells [22]. Moreover, PPARβ/δ inhibition promoted the gene and protein expression of matrix metalloproteinase 9 (MMP-9) and increased the adhesion of mouse melanoma B16F10 to endothelial cells leading to enhanced motility and invasiveness, which are crucial for melanoma metastasis. Furthermore, the protective role of PPARβ/δ signaling in the development of melanoma progression and metastasis was demonstrated in vivo [22].

The role of PPARα in melanomagenesis is still not fully elucidated. PPARα is mainly expressed in adipocytes, heart and skeletal muscles, and gastrointestinal tract tissues [24]. However, this receptor was also detected in cancer cells, including melanoma (SK-MEL-30 and WM-115 cell lines) [38]. The involvement of PPARα in carcinogenesis is discussed in the field of metabolism of essential elements such as lipids, glucose, and amino acids, and modulation of the immune response [24]. There are no studies concerning the direct effect of PPARα on the proliferation of melanoma cells; however, clinical studies confirmed the beneficiary effect of multi-modal therapy, including agonists of PPARα, on melanoma progression [39].

PPARs are involved not only in melanoma cell proliferation but also in cell cycle regulation. Importantly, previous studies suggested that the antiproliferative activity of PPAR agonists rather resulted from cell cycle arrest than induction of apoptosis in melanoma cell lines [26]. On the contrary, Placha et al. reported that PPARƴ agonist could also exert its biological action on melanoma cells by induction of apoptosis [28]. Thus, further studies are necessary to clarify this issue.

The biological activities of PPARs in melanoma are summarized in Table 1.

### 3.1. PPARs’ Ligands in Melanoma Treatment

Despite several in vitro and in vivo studies revealing the beneficiary potential of PPARs ligands in the inhibition of melanoma initiation, progression, and metastasis, clinical studies did not bring such optimistic conclusions in the sole use of PPAR ligands in melanoma therapy. However, so far, only a few studies have evaluated PPAR agonist in the treatment of melanoma. Reichle et al., in a phase II clinical trial, evaluated the efficacy of pioglitazone and rofecoxib combined with sequentially added angiostatic chemotherapy for 19 patients with stage IV melanoma [47]. The median progression-free survival was 2.8 months. Importantly, one patient achieved complete remission, and one patient partial remission. According to the authors, treatment with a PPARƴ agonist and cyclooxygenase-2 (COX-2) inhibitor might increase the susceptibility of melanoma cells to chemotherapy by up-regulating pro-apoptotic mechanisms. Since tumor-promoting inflammation is a typical feature of cancer, pioglitazone seems to be a promising agent due to its ability to decrease plasma C-reactive protein (CRP) levels by inhibiting interleukin 1 (*IL1*) expression. Reichle et al. demonstrated in the melanoma group that patients, who achieved a reduced plasma CRP level, improved progression-free survival [47]. In another study, combined therapy, including pioglitazone, etoricoxib, low-dose trofosfamide, and temsirolimus for stage IV melanoma, was shown to control both metastatic growth in cutaneous and uveal melanoma [48]. Currently, a phase II clinical trial is conducted in patients with advanced melanoma for whom monotherapy with Pembrolizumab or Nivolumab would be recommended according to the stage of the disease. The study aims to evaluate whether adding metformin or rosiglitazone, a PPARy agonist, will act synergistically with anti-PD-1 monoclonal antibody and compare if the response rate will be higher than with PD-1 monoclonal antibody [49].

### 3.2. PPARs in the Immune Response to Melanoma

Melanoma is considered one of the most immunogenic tumors. Possible mechanisms allowing melanoma to escape from immune control include:

- defective recognition of melanoma cells leading to inadequate activation of melanoma infiltrating lymphocytes,
- defective expression of immune checkpoint receptors,
- up-regulation of immune checkpoint ligands programmed cell death ligand 1 (PD-L1) and PD-L2 resulting in inhibition of T cell function,
- release of pro-apoptotic molecules by melanoma cells,
- up-regulation of immune suppressive populations, e.g., myeloid-derived suppressor cells (MDSCs), regulatory T cells (Tregs)
- release of pro-apoptotic molecules by melanoma cells [50],
- dysfunction of antigen processing and presentation [51].

Previous studies suggested that PPARs might be involved in the regulation of some of those processes in melanoma [24]. The PPARγ signaling pathway has recently been shown to control MDSCs expansion and T-cell proliferation. It was reported that genetic ablation of *LAL* gene led to the inactivation of PPARγ, increased MDSCs, and decreased T cell population, resulting in inflammation. Zhao et al. demonstrated that activation of PPARγ by 9-HODE impaired stimulatory effects of MDSCs on tumor growth and metastasis in *LAL*^−/−^ mice. A similar result was observed in the proliferation and migration of tumor cells in vitro [52].

Wu et al. investigated the antitumor effect of PPARγ antagonist GW9662 and anti-PD-L1 (aPD-L1) immunotherapy in a B16 murine melanoma [53]. Mice were divided into the following groups: treated with vehicle only (control), aPD-L1 alone, GW9662 alone, or a combination of PD-L1 and GW9662. However, gender differences were revealed in the clinical effect. The tumor size was reduced in female mice treated with aPD-L1, but the survival rate did not improve. GW9662 did not demonstrate any effect, whereas combined therapy of GW9662 and aPD-L1 decreased tumor growth and increased the survival rate. However, the combined treatment did not inhibit tumor growth in male mice. The inhibitory effect on tumor growth was observed only in the male group treated with aPD-L1. These findings follow clinical observation demonstrating worse and more aggressive course of melanoma in male patients [54]. Considering the positive correlation between obesity and melanoma progression, the authors compared results between obese and lean mice. In obese female mice, the effect was less pronounced than in lean females, whereas no reaction to the treatment was observed in obese male mice [53]. These findings might help provide the optimal immunotherapy for patients with melanoma.

Further studies defining the role of PPARs in the regulation of immune response in melanoma are necessary and might have clinical implications in the future.

## 4. PPARs and the Kynurenine Pathway

### 4.1. The Kynurenine Pathway

**Figure 2:**
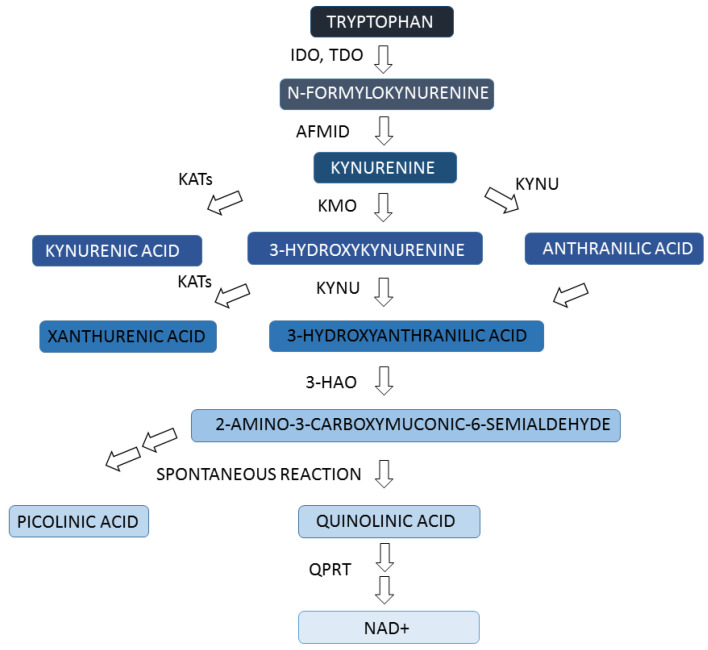
Simplified scheme of kynurenine pathway. The selected tryptophan metabolites and enzymes are shown in the scheme. AFMID—kynurenine formamidase, 3-HAO—3-hydroxyanthranilate 3,4-dioxygenase, IDO—indoleamine-2,3-dioxygenase, KATs—kynurenine aminotransferases, KMO—kynurenine-3-monooxygenase, KYNU—kynureninase, NAD—nicotinamide adenine dinucleotide, QPRT—quinolinate phosphoribosyl transferase, TDO—tryptophan 2,3-dioxygenase..

The kynurenine pathway is a major pathway of tryptophan metabolism (Figure 2). Approximately 95% of dietary tryptophan is metabolized in this pathway leading to nicotinamide adenine dinucleotide (NAD+) production. Indoleamine 2,3-dioxygenase (IDO) and tryptophan 2,3-dioxygenase (TDO) are considered rate-limiting enzymes of the kynurenine pathway catalyzing the first reaction of tryptophan conversion. Gene analysis revealed that melanoma expresses all genes coding enzymes of the kynurenine pathway (Figure 4). Kynurenine is a key metabolite of the kynurenine pathway, which is metabolized by kynurenine aminotransferases (KATs) to kynurenic acid or by kynurenine monooxygenase (KMO) to 3-hydroxykynurenine. There are four isoforms of KAT coding by *KYAT1*, *AADAT*, *KYAT3*, and *GOT2* genes. KATs are also involved in the synthesis of xanthurenic acid. In the main pathway, kynureninase (KYNU) catalyzes the conversion of 3-hydroxykynurenine to 3-hydroxyanthranilic acid but also the transformation of kynurenine to anthranilic acid. In the following steps, 3-hydroxyanthranilic acid is metabolized by 3-hydroxyanthranilate 3,4-dioxygenase (3HAO) to 2-amino-3-carboxymuconic acid-6-semialdehyde. Quinolinic acid, a product of a non-enzymatic reaction, is a direct substrate for the synthesis of NAD+ catalyzed by quinolinate phosphoribosyltransferase (QPRT) [55].

Importantly, all tryptophan metabolites possess the biological activity and play a role in various physiological and pathological processes. However, this review will focus on the potential role in carcinogenesis, cancer progression, and metastasis. Tryptophan metabolites may exert direct and indirect effects on melanoma progression and metastasis.

Tryptophan metabolites modulate the immune response, which may be crucial in cancer initiation and progression. Picolinic acid and kynurenic acid have anti-inflammatory activity, whereas kynurenine, 3-hydroxykynurenine, 3-hydroxyanthranilic acid, and quinolinic acid possess pro-inflammatory properties [55]. However, tryptophan metabolites also modify the immune response to cancer cells. Previous studies suggested that kynurenic acid regulated the immune response in melanoma via the interaction with IL-10, IL-7, and TNF receptor superfamily member 12A (*TNFRSF12A*) [56,57,58], whereas anthranilic acid might regulate the Th1/Th2 regulatory conversion by interaction with the IL-12-related and the Toll-like receptor (TLR) signaling pathways [58]. The role of 3-hydroxyanthranilic acid has not been fully revealed. Fallarino et al. reported that 3-hydroxyanthranilic acid induced selective apoptosis of Th1 [59]; however, other studies showed enhanced apoptosis of T-, B- and natural killer cells (NK) in response to this tryptophan metabolite [60]. 3-hydroxykynurenine, 3-hydroxyanthranilic acid, and quinaldic acid inhibited the proliferation of allogeneic T cells and suppressed Th1 cells [55]. Additionally, kynurenine is a potent immunosuppressive agent promoting the differentiation of Treg cells and preventing the differentiation of cytotoxic T cells, which provides an immunologically privileged microenvironment for cancer cells [61].

Importantly, tryptophan metabolites also directly affect cancer cells, including melanoma. Kynurenine significantly inhibited the proliferation of melanoma A375, SK-MEL-3, and RPMI-7951 cells modulating the protein expression of cell cycle regulators [62]. Surprisingly, although the antiproliferative effect of kynurenic acid was previously reported in colon cancer, renal cancer, and glioblastoma cell lines [63,64,65], kynurenic acid exerted only a slightly inhibitory effect on DNA synthesis in melanoma SK-MEL-3 cells but not in other melanoma cell lines [62]. Unfortunately, a limited number of publications have focused on the biological effects and molecular interactions of other tryptophan metabolites in melanoma. However, previous studies revealed that tryptophan metabolites exerted a biological effect on different types of cancer and might be involved in carcinogenesis. Therefore, it cannot be excluded that similar mechanisms may occur in melanoma. Although the direct effect of anthranilic acid on melanoma cell proliferation has not been studied so far, its elevated concentration in peritoneal lavage was reported in advanced gastric cancer [66]. Additionally, analogs and derivatives of anthranilic acid possess anticancer activity inhibiting the mitogen-activated protein kinase (MAPK) signaling pathway and inducing apoptosis of cancer cells [67]. Gan et al. [68] revealed that 3-hydroxyanthranilic acid might also have anticancer properties. It sensitized hepatocellular carcinoma cells to sorafenib decreasing the activity of Akt kinase and inducing apoptosis of cancer cells. The molecular mechanism of the activity of xanthurenic acid has not been studied so far; however, its concentration in serum from patients diagnosed with non-small cell lung cancer was significantly decreased compared to healthy control [69]. Although a limited number of studies focused on the biological activity of 3-hydroxykynurenine towards cancer cells, the involvement of KMO, the enzyme catalyzing the reaction of kynurenine conversion to 3-hydroxykynurenine, in carcinogenesis and cancer progression has been reported [70]. The overexpression of *KMO* was reported in breast cancer, colorectal cancer, and hepatocellular carcinoma [70]. Importantly, the overexpression of *KMO* led to the expression of various genes involved in the proliferation, survival, invasiveness, and metastasis of cancer cells [71]. Similarly, quinolinic acid stimulated the expression of genes involved in cancer cell proliferation and survival via the MAPK signaling pathway [72].

### 4.2. The PPAR Signaling Pathway and the Kynurenine Pathway

**Figure 3:**
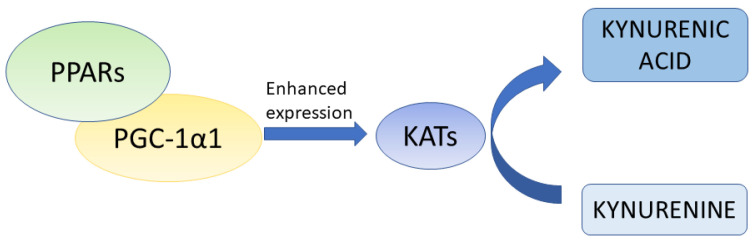
The simplified scheme of interaction between the PPAR pathway and the kynurenine pathway in skeletal muscles [73]. PGC-1α1 may interact with various transcription factors, including PPARs, and plays a crucial role in the interaction between the PPAR pathway and the kynurenine pathway. In skeletal muscles, PGC-1α1 induces the expression of KATs, which convert kynurenine to kynurenic acid. KATs—kynurenine aminotransferases, PGC-1α1—peroxisome proliferator-activated receptor gamma coactivator-1 alpha 1, PPARs—peroxisome proliferator-activated receptors.

Previous studies confirmed the functional link between PPARs and the kynurenine pathway in skeletal muscles. Agudelo et al. [73] revealed that activation of the PPARα/δ pathway in muscles led to overexpression of KATs, which converted kynurenine to kynurenic acid (Figure 3). The key element in this crosstalk was peroxisome proliferator-activated receptor gamma coactivator-1 alpha 1 (PGC-1α1). The interaction between the PGC-1α1/PPARα/δ pathway and the kynurenine pathway in skeletal muscles proved the hypothesis that physical exercises might modify mood and, more importantly, physical activity might be considered a new therapeutic approach to the treatment of depression [73]. Unfortunately, the relationship between the PPAR pathway and the kynurenine pathway in tissues other than skeletal muscles in physiological or pathological conditions has not been revealed.

## 5. Potential Interaction of the PPAR Signaling Pathway and the Kynurenine Pathway in Melanoma

**Figure 4:**
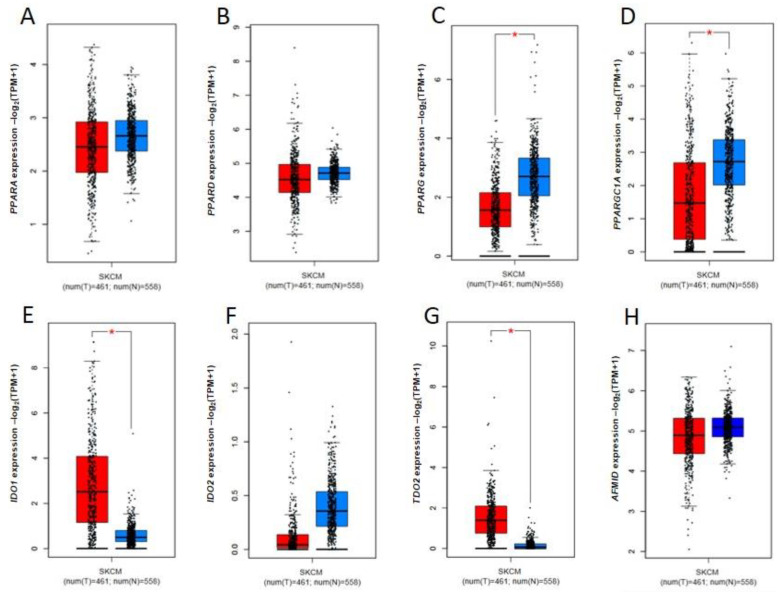

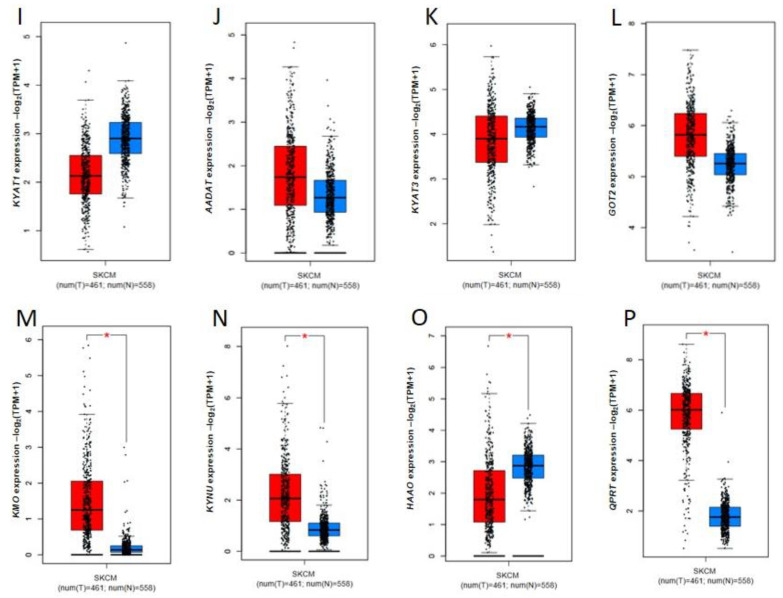
Expression pattern of genes coding proteins involved in the PPAR pathway ((**A**) *PPARA*, (**B**) *PPARD*, (**C**) *PPARG*, (**D**) *PPARGC1A*) and enzymes of the kynurenine pathway ((**E**) *IDO1*, (**F**) *IDO2*, (**G**) *TDO2*, (**H**) *AFMID*, (**I**) *KYAT1*, (**J**) *AADAT*, (**K**) *KYAT3*, (**L**) *GOT2*, (**M**) *KMO*, (**N**) *KYNU*, (**O**) *HAAO*, (**P**) *QPRT*) in human skin cutaneous melanoma (SKCM) (red; N = 461) in comparison to normal control tissue (skin) (blue; N = 558). Gene expression data from the TCGA and GTEx datasets were retrieved and analyzed by GEPIA2 [79]. Differences in gene expression levels were statistically assessed using ANOVA. * *p* < 0.01 and fold-change threshold (|Log2FC| cutoff) of 1. TPM, transcripts per million.

Gene expression data revealed that there might be a possible link between the PPAR signaling pathway and the kynurenine pathway in melanoma. Although PPAR isoforms have been previously related to cancer progression and some studies considered PPAR as a negative prognostic marker [74,75,76,77], surprisingly, *PPARG* is down-regulated in human skin cutaneous melanoma (SKCM) in comparison to corresponding normal tissue (Figure 4C). However, there were no significant differences in the expression profile of other *PPAR* genes. Indeed, the involvement of the PPAR pathway in cancer promotion and progression is ambiguous and has been recently deeply reviewed by Wagner and Wagner [77]. The data obtained from the Human Protein Atlas confirmed that PPARγ is weakly expressed in melanoma tissue [78]. Notably, *PPARGC1A* gene coding PGC-1α is also down-regulated in melanoma (Figure 4D). On the other hand, various kynurenine pathway-related genes are overexpressed in SKCM compared to healthy control, including *IDO1*, *TDO*, *KMO*, *KYNU*, and *QPRT* (Figure 4E,G,M,N,P). Surprisingly, the expression of genes coding KATs, key elements of direct interaction between the PPAR pathway and the kynurenine pathway, is not modified. Importantly, previous studies reported that mutations in genes coding KATs in melanoma were correlated with a reduced survival rate [58]. Unfortunately, this data does not verify whether overexpression of the kynurenine pathway-related genes in melanoma is involved in the increased demand for energy sources (NAD+) in the cancer tissue or the production of tryptophan metabolites influencing tumor tissue or tumor microenvironment.

Previous studies revealed the functional connection between the PPAR pathway and induction of the expression of KATs’ genes via PGC-1α activation [73]. Gene expression data suggest some link between the PPAR and the kynurenine pathways in melanoma; however, further studies are necessary to verify whether the functional connection between these pathways, similar to those in muscles, is also present in melanoma or melanoma environment. It cannot be excluded that the PPAR pathway and the kynurenine pathway may interact with each other in melanoma in different manners.

**Figure 5:**
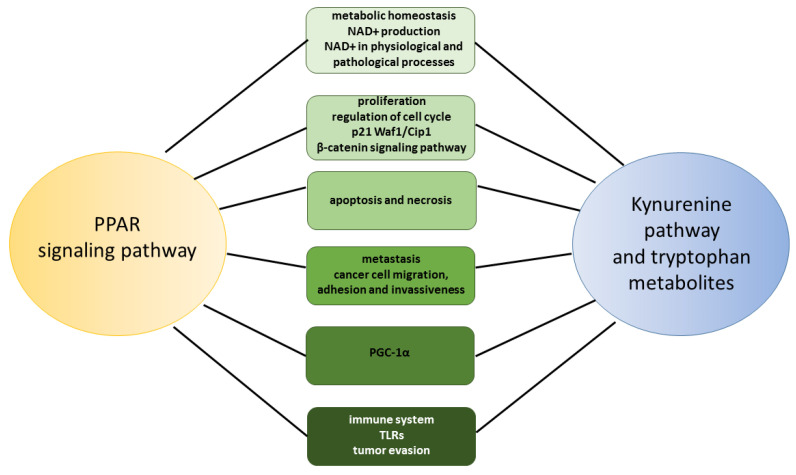
Scheme of potential interactions between the PPAR signaling pathway and the kynurenine pathway.

Although no studies confirm the direct link between the kynurenine pathway and the PPAR pathway in melanoma, some biological effects of tryptophan metabolites are similar to those observed after the activation of PPARs. In this review, we will focus on selected cellular processes in cancer cells, including melanoma, to identify the fields where the kynurenine pathway and the PPAR pathway may interact (Figure 5).

### 5.1. Metabolism

Metabolic plasticity is a characteristic feature of cancer cells that easily adapt to nutrient restrictions or other environmental conditions, including hypoxia or pH changes [80,81]. On the other hand, up-regulation and activation of oncogenic pathways in cancer cells lead to disruption in cytosolic and mitochondrial metabolic pathways [82,83].

The main route of the kynurenine pathway is involved in the production of NAD, an essential redox cofactor. Appropriate NAD level is necessary to maintain intracellular redox homeostasis; however, it is also crucial for all biological processes dependent on NAD-related enzymes (i.e., poly-ADP-ribose polymerases (PARPs), sirtuins, NAD glycohydrolase), including DNA plasticity and repair, cell adaptation to stress, cell death, and proliferation, or immune response [84,85]. Most of these processes are disrupted in melanoma; thus, regulation of NAD production in cancer cells is crucial for cancer promotion, progression, and metastasis.

NAD is synthesized from tryptophan via the kynurenine pathway. Quinolinic acid is considered a precursor of NAD de novo synthesis [86]. It should be noted that *QRPT*, coding a final and rate-limiting enzyme of kynurenine pathway, is overexpressed in melanoma (Figure 4P). This observation suggests that the kynurenine pathway in melanoma is directly involved in NAD production to meet the growing energy demands of cancer cells.

Despite no studies focusing on PPARs and NAD in melanoma, the functional network between PPAR pathway, kynurenine pathway, and NAD has been previously reported in acute kidney injury [87]. Functional interaction between PGC-1α and NAD in skin physiology has also been recently studied. PGC-1α is a transcriptional coactivator involved in the regulation of metabolism and mitochondrial function in several tissues and organs [88,89]. Wong et al. [90] reported that PGC-1α plays an essential role in NAD homeostasis during skin aging. NAD is involved in the regulation of cell growth under physiological stress conditions in the skin interacting with the p53/p21 signaling pathway. Importantly, PGC-1α sustains the level of NAD, which is crucial for epidermal repair [90].

### 5.2. PGC-1α

PPARs interact with coactivators, such as PGC-1 family: PGC-1α, PGC-1β, and PGC-1-related coactivator (PRC) [91]. PGC-1α is a critical transcriptional coactivator involved in the regulation of mitochondrial metabolism; however, it controls various tissue-specific processes, including angiogenesis, adipogenesis, glucose metabolism, and cell survival, by interaction with transcription factors and nuclear receptors [92]. PGC-1α is involved in mRNA splicing since it possesses the characteristic RNA recognition motif and arginine/serine domain [91]. Importantly, PGC-1α interacts not only with PPARs but also with other nuclear receptors, including estrogen receptors (ERs) and nuclear respiratory factor (NRF) 1 and 2 [93].

PGC-1α plays an important role in the regulation of physiological processes within the skin. A recent study indicated the crucial role of PGC-1α in epidermal repair [90]. Additionally, Shoag et al. reported that this transcriptional coactivator regulated the production of melanin interacting with microphthalmia-associated transcription factor (MITF) [94].

The role of PGC-1α in carcinogenesis remains controversial. Due to various processes regulated by PGC-1α, its role in cancer depends on tissue requirements and, in most cases, is still unclear. Similarly, the expression pattern of PGC-1α is tumor-type dependent. There is no unequivocal expression trend in melanoma since previous studies reported increased and decreased *PGC1A* gene expression [92]. Additionally, the role of PGC-1α in melanoma has not been fully revealed. PGC-1α, inducing oxidative metabolism, stimulates the proliferation and survival of melanoma cells, but on the other hand, it inhibits invasiveness and melanoma metastasis [95,96,97]. It was also reported that this transcriptional coactivator stimulated melanogenesis by interaction with MITF [98]. Importantly, MITF/PGC-1α positive melanomas are more resistant to ROS-induced apoptosis due to enhanced mitochondrial oxidative metabolism and detoxifying properties of cancer cells [98,99]. PGC-1α may also be involved in the chemoresistance of melanoma cells [89]. PGC-1α increases the expression of ROS detoxifying genes favoring tumor cell survival [99].

Previous studies confirmed that activation of the PGC-1α/PPAR pathway might benefit the immune response to cancer cells [100]. Additionally, it should be noted that microenvironmental conditions might modify the biological effect of the activation of PGC-1α in other types of cancer [92]. It cannot be excluded that this mechanism may also be applied to melanoma.

PGC-1α is the direct link between PPARs and the kynurenine pathway, as previously confirmed in skeletal muscles [73]. Allison et al. [101] reported that overexpression of PGC-1α was correlated with overexpression of all KAT isoforms. Thus, the interaction between PGC-1α and the kynurenine pathway has both functional and genetic levels. Although this dependence has not been confirmed in melanoma to date, it cannot be excluded that similar interactions between tryptophan metabolites and PGC-1α are also present in melanoma.

### 5.3. Cellular and Molecular Effects

#### 5.3.1. Proliferation

The biological role in cancer cell proliferation of PPARs has been extensively studied, and PPARγ activity in melanoma is especially well-documented. Various PPARγ agonists exert antiproliferative activity towards melanoma in vitro and in vivo [27,30,31,32]. Previous studies revealed that PPARƴ agonists, including troglitazone, rosiglitazone, and 15d-PGJ2, inhibited the proliferation of human melanoma cell lines in a dose-dependent manner and induced cell cycle arrest, whereas no effect was observed in melanoma cells exposed to PPARα agonist WY-14643 [26]. Similar results were obtained by Freudlsperger et al., who reported the inhibitory effect of rosiglitazone, pioglitazone, ciglitazone, and troglitazone on melanoma cell lines derived from both primary (UISO-Mel6, G361) and metastatic melanoma (MV3, MeWo, Lox, Fem-X1) [27]. Among all PPARƴ agonists, the most potent antiproliferative effect was demonstrated by ciglitazone [27]. The authors observed that glitazones in a concentration higher or equal to 30 mmol/L exerted an inhibitory effect on melanoma cell lines, but in lower concentration (3 mmol/L) slightly stimulated cell proliferation. Therefore, it was suggested that the dose-dependent effect of glitazones should be considered in planning in vivo studies to avoid possible stimulatory effect on malignant melanoma [27].

Importantly, PPARγ seems to play an important role not only in the regulation of the proliferation of melanoma cells but there is also the direct link to the tumor-stroma interactions [35]. Paulitschke et al. reported that 15d-PGJ2, one of the PPARγ agonists, affected the proliferation of tumor-associated fibroblasts [35]. Interactions with tumor-associated fibroblasts and endothelial cells suggest that PPARγ is involved in melanoma proliferation and angiogenesis, leading to melanoma progression and metastasis [35]. Even though the beneficial effect of PPARγ in cancer has been confirmed in several studies, the recent one brings a new perspective to the matter. Peng et al. [32] reported that irreversible PPARγ antagonist MM902 inhibited the proliferation of cancer cells, including melanoma LOX-IMVI and MALME-3M cells. Additionally, the beneficiary effect of PPARγ antagonist was also confirmed in in vivo studies, where MM902 inhibited tumor growth in the mouse xenograft model of melanoma [32].

The role of PPARα and PPARβ/δ in cancer cell proliferation is more controversial [22]. Borland et al. [40] reported that PPARβ/δ had similar antiproliferative activity to PPARγ in melanoma in vitro and in vivo and could have a beneficial effect in the chemoprevention of primary and metastatic melanoma. Moreover, disruption of PPARβ/δ signaling pathway by either antagonist or gene knock-down led to melanoma progression [22], whereas activation or overexpression of PPARβ/δ inhibited cell cycle progression in the G2/M phase of melanoma cells in ectopic xenografts [40].

The molecular studies addressed various molecular mechanisms for the antiproliferative activity of PPAR agonists, including regulation of the cell cycle, signaling pathways, and induction of cell death [23,34,35,40,102,103].

Importantly, tryptophan metabolites also affect cancer cell proliferation. Although there is a limited number of studies focused on the direct effect of kynurenines on melanoma cells, the involvement of this group of substances in cancer promotion and progression has been reported. Kynurenine has a significant antiproliferative activity toward melanoma cells [104]. This tryptophan metabolite at a concentration of 1 pM inhibited proliferation and DNA synthesis in melanoma A375 cells; however, the strongest effect was observed in millimolar concentrations [104]. On the other hand, the effect of kynurenine on carcinogenesis has been discussed. Thaker et al. reported that this tryptophan metabolite stimulated the proliferation of colon cancer HCT116 cells in vitro via activation of the β-catenin pathway [105]. Interestingly, another tryptophan metabolite, kynurenic acid, inhibited the proliferation of various cancer cell lines in vitro, including colon cancer, renal cancer, and glioblastoma [63,64,65,106], but it did not affect DNA synthesis of melanoma A375 and RPMI-7951 cells [104]. Kynurenic acid in millimolar concentrations inhibited the DNA synthesis and metabolic activity of only one tested melanoma cell line, SK-MEL-3 [62], which might suggest that the biological activity of this tryptophan metabolite is not cell type dependent but rather genetic differences might have an impact on the antiproliferative activity of kynurenic acid.

#### 5.3.2. Cell Cycle Regulation—p21 Waf1/Cip1

Previous studies confirmed the involvement of PPARs in the cell cycle regulation of cancer cells. Anticancer activity of PPARγ is based on cell cycle arrest rather than cell death induction [1]. The majority of studies confirmed the critical role of cyclins, retinoblastoma (Rb), p21 Waf1/Cip1, and β-catenin in PPAR-dependent cell cycle arrest [26,29,30,107,108]. Although previous studies indicated only the involvement of PPARγ in the regulation of cell cycle proteins in melanoma, studies conducted on other types of cancer confirmed that all PPAR isoforms might have a similar biological effect [109,110].

A similar biological effect was observed in melanoma cells in vitro after exposure to tryptophan metabolites [62]. Kynurenine and kynurenic acid increased the protein expression of cell cycle inhibitors p21 Waf1/Cip1 and p27 Kip1 in melanoma SK-MEL-3 cells [62]. Importantly, kynurenine exerted a more potent stimulatory effect. The involvement of p21 Waf1/Cip1 in the antiproliferative activity of kynurenic acid has been previously reported in colon cancer HT-29 cells [111]. Furthermore, kynurenine and kynurenic acid inhibited the protein expression of cyclin-dependent kinase (CDK) 4 and phosphorylation of Rb in melanoma cell lines [62,104].

Unfortunately, there are limited data concerning the biological activity of other metabolites of the kynurenine pathway toward melanoma. However, it should be noted that overexpression of p21 Waf1/Cip1 was also observed in cancer cells exposed to derivatives of anthranilic acid [112,113].

The role of tryptophan metabolites in the activation of the β-catenin signaling pathway has not been fully revealed. Although neither kynurenine nor kynurenic acid affected the protein expression of β-catenin in melanoma SK-MEL-3 cells [62], the stimulatory effect was observed in colon cancer HT-29 cells exposed to millimolar concentrations of kynurenic acid [106]. Similarly, kynurenine and quinolinic acid activated β-catenin leading to increased proliferation of colon cancer cells and tumor growth [105].

#### 5.3.3. Cell Death

The PPAR signaling is also involved in cell death of cancer cells, including melanoma. However, the role of particular isoforms of PPAR in cell death induction in melanoma has not been fully revealed. Importantly, there is a strict dependency between PPAR isoforms. Maggiora et al. showed that an increase in PPARα protein level with a simultaneous decrease in PPARβ/δ protein level led to apoptosis of cancer cells in response to linoleic acid [114]. On the contrary, overexpression of *PPARβ/δ* induced apoptosis in hepatocellular carcinoma Hep2G cells [115]. Thus, the role of PPARs in the induction of apoptosis seems to be tissue- or cell-type-dependent. Similarly, previous studies reported the functional link between the activation of PPARα and cell death. Kong et al. [116] revealed that activation of PPARα by fenofibrate led to apoptosis of colon cancer cells. Previous studies suggested that activation of PPARγ is related to the inhibition of proliferation of cancer cells rather than induction of cell death [26,27]. However, several studies reported the direct involvement of PPARγ in apoptosis, including in melanoma cells [41,42,117]. Rosiglitazone activating PPARγ induced apoptosis in A375 cells by decreasing Bcl-2 level while increasing p53 protein expression [41].

Similarly, the role of tryptophan metabolites in cell death induction is unclear. Our group revealed that kynurenic acid at a concentration of 5 mM induced apoptosis in melanoma A375 cells, but a similar effect was not observed in RPMI-7951 cells representing metastatic melanoma. Significantly, kynurenic acid also stimulated necrosis in melanoma A375 cells, suggesting that pro-apoptotic activity was not a target molecular mechanism of this tryptophan metabolite [104].

There is only one report regarding the effect of kynurenine on melanoma cell death. Kynurenine stimulated necrosis in melanoma A375 cells but not in metastatic melanoma RPMI-7951 cells [104].

#### 5.3.4. Metastasis

Metastasis is a multistep process resulting from the accumulation of genetic and epigenetic alternations, which is associated with poor prognosis for melanoma patients. PPARs are involved in all processes of melanoma metastasis, including epithelial-mesenchymal transition (EMT), migration, adhesion, invasiveness, and modifications of the tumor microenvironment [118]. Importantly, previous studies revealed some controversies. The results are contradictory; thus, further studies are necessary to verify the hypothesis of the involvement of particular isoforms of PPAR in metastasis and give a clear answer regarding their positive or negative role in this process. The majority of studies underlined the crucial role of PPARβ/δ in melanoma metastasis. It was reported that PPARβ/δ inhibition increased melanoma cell migration and invasiveness in vitro and promoted lung metastasis in vivo [22]. On the contrary, PPARβ/δ has been previously associated with promotion of the aggressive phenotype of melanoma. Activation of PPARβ/δ in highly metastatic melanoma cell lines resulted in the increased migration mediated by overexpression of *SNAIL* [46].

Anti-migratory effect of fenofibrate towards melanoma cell lines has also been previously reported confirming the involvement of PPARα in cancer metastasis [43]; however, the role of PPARα in melanoma metastasis is not fully elucidated. Stebbins et al. [119] reported that inhibition of PPARα by NXT629 decreased lung metastasis of B16F10 cells in mice model.

The role of PPARγ in melanoma metastasis is unclear; however, it might be suggested that the role of this receptor depends on the target: melanoma cell or tumor microenvironment. Previous studies revealed that activation of PPARγ by cloxiquine resulted in decreased metastasis of melanoma cells in a mice model [120]. Although the majority of in vitro studies confirmed the beneficiary role of PPARγ agonists in cancer chemoprevention and suggested their antiproliferative and anti-metastatic properties, the results of clinical studies are not so optimistic [1,121,122,123]. It was suggested that the potential impact on the tumor environment might be crucial. Rostiglitazone, the PPARγ agonist, induced the expression of cytokines, chemokines, and angiogenesis-stimulating factors modifying the tumor microenvironment to favor metastasis [123].

Similarly, the role of the kynurenine pathway in metastasis has not been fully revealed. Previous studies reported that the effectiveness of kynurenine and kynurenic acid towards melanoma cells depended on the stage of melanoma progression. Tryptophan metabolites were less effective towards metastatic melanoma RPMI-7951 cells than primary melanoma A375 cells [104]. The role of kynurenine and kynurenic acid on the invasiveness of melanoma cells is more controversial. Although these tryptophan metabolites did not affect the migration of A375 and RPMI-7951 cells, kynurenine stimulated the migration of melanoma SK-MEL-3 cells and a similar effect was observed in UVB-treated SK-MEL-3 cells in response to kynurenic acid [62]. The molecular mechanism of these interactions was not revealed. However, the stimulatory effect of kynurenine on cancer cell migration was reported previously. Kynurenine stimulated the migration and metastasis of lung cancer 95D cells by increasing the remodeling of the extracellular matrix [124]. The immunosuppressive activity of kynurenine should also be underlined in this discussion.

### 5.4. Interactions with the Immune System

Tumor cells interact with the immune system during melanomagenesis. Immune evasion seems to be the greatest challenge in melanoma therapy. Melanoma immune escape results from progressive exhaustion of the immune system by chronic stimulation and specific mechanisms of tumor cells leading to counteract the antigenic recognition [50]. The molecular strategies of immune escape include:

- dysregulation of the expression of cell signaling molecules on the effector cells,
- the release of melanoma-derived soluble factors involved in immune suppression, including vascular endothelial growth factor (VEGF), tumor necrosis factor (TNF), transforming growth factor β (TGF-β), IL-1, IL-6, IL-10, prostaglandin E2 (PGE2),
- variability of the tumor antigen expression,
- polarization of Th1 cells [125].

An important mechanism in melanoma progression is the dysregulation of antigen processing and presentation. A characteristic feature of melanoma cells is the heterogeneity of the antigenic repertoire necessary to evade immune system control [126]. Importantly, actively proliferating melanoma cells decrease the presentation of the major histocompatibility class-I (MHC-I) complex to effector CD8+ T cells. Additionally, the defective activity of dendritic cells (DC) and reduced cytotoxicity mediated by CD8+ T cells result in an impaired immune response to melanoma cells [125].

Previous studies confirmed that the kynurenine pathway is involved in the immune response. The majority of studies focused on the IDO1 activity. Kai et al. [127] reported that IDO1 is crucial for normal cytotoxicity of natural killer (NK) cells against cancer cells, including melanoma [128,129]. On the contrary, Frumento et al. revealed that IDO1 inhibited the proliferation of NK and T cells [130]. In addition, overexpression of *IDO1* in monocytes and low activity of IDO1 in response to INFγ correlated with worse outcomes in melanoma patients [131]. It should be noted that not only IDO1 activity but also the biological properties of kynurenine, the product of the enzymatic reaction, is associated with dysregulation of the immune response. Previous studies indicated the regulatory function of kynurenine in NK activity via the signal transducer and activator of transcription (STAT) 1 and 3 signaling pathways [132]. Moreover, the kynurenine pathway enzymes may initiate tolerogenesis in a DC-dependent and DC-independent manner [128,133].

Importantly, TLRs could be another common point between PPARs and kynurenine pathway. TLRs belong to the family of pattern recognition receptors [134]. Importantly, TLRs are mainly expressed in keratinocytes (*TLR1*, *TLR2*, *TLR3*, *TLR4*, *TLR5*, *TLR6*, *TLR9*) and melanocytes (*TLR2*, *TLR3*, *TLR4*, *TLR5*, *TLR7*, *TLR9*, *TLR10*) within the human skin [135]. Ligation of TLR resulted in various innate and adaptive immune responses via activation of the NF-κB signaling pathway and induction of INF [134]. It should be underlined that TLRs are also expressed in cells of the immune system, including monocytes, macrophages, and dendritic cells [136]. However, previous studies confirmed TLRs’ involvement in carcinogenesis, tumor microenvironment modifications, and cancer immune escape. TLR-4 was associated with the up-regulation of pro-inflammatory cytokines in melanoma cells [137]. Interestingly, carcinogenesis was less common in TLR-4-deficient mice [138]. Previous studies revealed that PPARγ agonists might inhibit TLRs activity and regulate the expression of *TLR4* gene [139,140,141]. Additionally, the anti-inflammatory activity of fenofibrate was enhanced by the inhibition of the TLR-4 signaling pathway in melanoma [137].

Importantly, activation of TLR led to an increase of IDO protein expression in dendritic cells leading to inhibition of T-cell proliferation [142]. Similarly, TLR-dependent immunosuppression of bone marrow-derived mesenchymal stem cells was mediated by kynurenines produced by IDO1 [143]. Activation of TLR-2, TLR-3, TLR-4, TLR-7/8, and TLR-9 resulted in an increased level of kynurenine in human peripheral monocytes, whereas activation of TLR-3 increased the level of kynurenic acid and quinolinic acid [136]. Previous studies underlined the role of kynurenine in immune suppression and evasion, leading to cancer cell survival [144,145].

### 5.5. Microbiota

Recent studies revealed that microbiome might play a role in carcinogenesis and response to cancer therapy [146,147]. Microbiome affects tumor cell metabolism and modifies the immune response. Previous studies suggested that skin microbiome might be involved in inflammatory and infectious skin diseases but also in skin cancer [147,148].

Changes in the composition of skin and gut microbiota were reported in melanoma patients. Moreover, the presence of specific types of bacteria was related to the stage of the disease progression [147,149,150]. Mizuhashi et al. reported that *Corynebacterium* was associated with advanced melanoma [149], whereas *Staphylococcus epidermidis* was suggested to have protective properties against melanoma cells [151]. It should be noted that not only skin microbiota but also gut microbiota and its metabolites may play a role in melanomagenesis and immune response to cancer cells [152,153]. Microbiome-derived metabolites may interact with PPARs modifying the energy metabolism and other cellular processes controlled by these receptors [154]. Short-chain fatty acids (SCFAs), including butyrate and propionate, stimulate the transcriptional activity of PPARs [155,156]. Importantly, bacterial lipopolysaccharide (LPS) is an exogenous ligand for TLR-4, and previous studies reported that PPARγ agonists regulated the expression of *TLR4* gene [140,141,157].

Importantly, the relationship between microbiome and the kynurenine pathway has been previously confirmed. Various bacterial phyla, including *Actinobacteria*, *Bacteroides*, *Firmicutes*, *Fusobacteria*, and *Proteobacteria*, metabolize tryptophan via the kynurenine pathway, producing the biologically active metabolites [158]. Previous studies confirmed a functional network between kynurenine, kynurenic acid, and PPARs [73]. In addition, there is a strong relationship between IDO1 and gut microbiota. Microbiota modifies the bioavailability of tryptophan and, therefore, dysregulates IDO1 activity and the kynurenine pathway. On the other hand, IDO1 may change the metabolism of gut microbiota and immune reactivity, inducing immunosuppression in the gastrointestinal tract [159]. Although most studies are focused on gut microbiota, the involvement of skin microbiota in the regulation of skin diseases, including melanoma, has been previously confirmed [147].

Considering the correlation between microbiota, PPARs, and the kynurenine pathway, skin and gut dysbiosis in melanoma patients may affect the activity of both metabolic pathways, the PPAR pathway and the kynurenine pathway. Although further studies are necessary, previous studies suggested that this functional interaction may directly and indirectly modify the tumor microenvironment and immune response to cancer cells.

## 6. Conclusions

The functional relationship between the PPAR signaling pathway, PGC-1α, and the kynurenine pathway was previously reported in skeletal muscles. However, some bioinformatics data and biological activity of PPAR ligands and tryptophan metabolites may suggest a potential involvement of these metabolic and signaling pathways in melanoma promotion, progression, and metastasis. It should be underlined that the potential cross-talk between the PPAR signaling pathway and the kynurenine pathway may refer not only to the direct biological impact on melanoma cells but also to the tumor microenvironment and the immune system. Previous studies revealed that the PPAR signaling pathway and the kynurenine pathway might be involved in the regulation of various processes in melanoma, including metabolism, proliferation, cell cycle regulation, cell death, and metastasis. Importantly, both signaling and metabolic pathways modify the immune response by direct impact on immune cells, production of cytokines, antigen presentation, and interaction with microbiome. Unfortunately, the involvement of the kynurenine pathway in melanomagenesis, melanoma progression, and metastasis has not been fully revealed. Therefore, the biological effects of tryptophan metabolites against other types of cancer may suggest new directions for further research. Determining the interactions between the PPAR signaling pathway and the kynurenine pathway in melanoma should be a priority, taking into consideration that human skin is constantly exposed to tryptophan metabolites, which are naturally synthesized in the skin and are present in various herbs and honey bee products used in skin care treatments [160,161]. Knowledge about the interaction between the PPAR pathway and the kynurenine pathway may contribute to introducing new chemopreventive agents or therapies against melanoma.

## Figures and Tables

**Table 1:** The biological activities of PPARs in melanoma.

PPAR Isoform	PPAR Ligand/PPAR Activity	Biological Effect	Research Model	Literature
**Cell cycle regulation**				
PPARβ/δ	overexpression	Cell cycle arrest; Decreased fraction of G1 and S phase; Increased fraction of G2/M phase	Human melanoma UACC903 cells	[40]
PPARγ	overexpression	Cell cycle arrest; Decreased fraction of G1 and S phase; Increased fraction of G2/M phase	Human melanoma UACC903 cells	[40]
PPARγ	Troglitazone (agonist)	Cell cycle arrest; Increased fraction of G1 phase	Human melanoma MM-201 cells	[26]
PPARγ	Ciglitazone (agonist)	Cell cycle arrest; Increased fraction of G0/G1 phase; Decreased expression of cyclin D1, Increased expression of p21 Waf1/Cip1 Reduced hyperphosphorylation of pRb	Human melanoma A375 cells	[30]
PPARγ	15d-PGJ2 (agonist)	Cell cycle arrest; Increased fraction of G_2_/M phase; Increased expression of p21 Waf1/Cip1; Increased expression and/or phosphorylation of p53	Human melanoma A375, M24met, and 1205Lu cells	[35]
PPARγ	Rosiglitazone (agonist)	Cell cycle arrest in G1 phase	Human melanoma A375 cells	[41]
PPARγ	Ciglitazone (agonist) 9-cis-retinoic acid (a retinoid X receptor (RXR) ligand)	Cell cycle arrest; Increased fraction of G0/G1 phase; Decreased fractions of S and G2/M phase	Mouse melanoma S91 cells	[42]
PPARγ	Troglitazone (agonist) Halofenate (selective modulator)	Decreased expression of cyclin D1; Increased expression of p21 Waf1/Cip1	Human melanoma MM96L cells	[29]
**Signaling pathways**				
PPARα	Fenofibrate (agonist)	Inhibition of Akt and extracellular signal-regulated kinase (ERK) l/2	Mouse melanoma B16F10 cells	[43]
PPARα	Fenofibrate (agonist)	Up-regulation of p38 MAPK	Mouse melanoma B16F10 cells	[44]
PPARγ	Troglitazone (agonist) Halofenate (selective modulator)	Down-regulation of β-catenin	Human melanoma MM96L cells	[29]
PPARγ	Rosiglitazone (agonist)	Down-regulation of the expression and phosphorylation of ERK 1/2	Human melanoma A375 cells	[41]
**Cell death**				
PPARα	Fenofibrate (agonist)	Sensitization of melanoma cells to proapoptotic drug staurosporine	Mouse melanoma B16F10 cells	[43]
PPARγ	overexpression	Increased apoptosis	ectopic xenografts derived from UACC903-Migr1 cells	[40]
PPARγ	Troglitazone (agonist)	No effect on apoptosis	Human melanoma MM-201 cells	[26]
PPARγ	Ciglitazone (agonist)	Induced apoptosis; Activation of caspase-9 cleavage of PARP	A375 melanoma tumor xenograft development in mice	[30]
PPARγ	Rosiglitazone (agonist)	Induced apoptosis; Increased expression of p53, Reduced expression of Bcl-2	Human melanoma A375 cells	[41]
PPARγ	rosiglitazone (agonist) T0070907 (inhibitor)	No effect on apoptosis and necrosis	Human melanoma WM4265.2-BrM1 and WM4265.2-BrM2 cells	[45]
**Migration, invasiveness, and metastasis**				
PPARα	Fenofibrate (agonist)	Inhibition of migration and colony formation	Mouse melanoma B16F10 cells and human melanoma SkMel188 cells	[43]
PPARβ/δ	10h (antagonist)	Increased motility and invasiveness; Increased expression of MMP9; Increased pulmonary extravasation of B16F10 cells	Mouse melanoma B16F10 cells C57BL/6 mouse model	[22]
PPARβ/δ	GW501516 (agonist)	Increased migration and invasion of A375SM cells, but not A375P cells; Increased expression of fibronectin and type I collagen; Increased expression of Snail in A375SM cells; Decreased expression of E-cadherin in A375SM cells	Human melanoma A375P and A375SM cells	[46]
PPARγ	15d-PGJ2 (agonist)	Decreased migration	Human melanoma A375 and M24met cells	[35]
PPARγ	polyunsaturated fatty acids/Activation by astrocytes	Pro-metastatic effect, Increased proliferation of metastatic melanoma cells	patient-derived xenografts; brain tropic melanoma cells; human melanoma WM4265.2, WM793, and WM1366 cells; mouse melanoma Yumm1.7 cells	[45]

## Data Availability

Not applicable.

## Abbreviations

3-HAO	3-hydroxyanthranilate 3,4-dioxygenase
AFMID	kynurenine formamidase
CDK	cyclin-dependent kinase
COX-2	cyclooxygenase-2
CRP	C-reactive protein
DC	dendritic cells
EMT	epithelial-mesenchymal transition
Ers	estrogen receptors
ERK	extracellular signal-regulated kinase
IDO	Indoleamine 2,3-dioxygenase
IL	interleukin
KAT	kynurenine aminotransferase
KMO	kynurenine-3-monooxygenase
KYNU	kynureninase
MAPK	mitogen-activated protein kinase
MDSCs	myeloid-derived suppressor cells
MHC-I	major histocompatibility class-I
MITF	microphthalmia-associated transcription factor
MMP	matrix metalloproteinase
mRNA	messenger RNA
NAD	nicotinamide adenine dinucleotide
NK	natural killer cells
NRF	nuclear respiratory factor
PARP	poly-ADP-ribose polymerase
PD-L1	programmed cell death ligand 1
PGC-1α	peroxisome proliferator activated receptor gamma coactivator-1 alpha
PGE2	prostaglandin E2
PRC	PGC-1-related coactivator
PPAR	Peroxisome proliferator-activated receptor
RXR	retinoid X receptor
QPRT	quinolinate phosphoribosyl transferase
Rb	retinoblastoma
SCFAs	Short-chain fatty acids
SKCM	skin cutaneous melanoma
TDO	tryptophan 2,3-dioxygenase
TGF-β	transforming growth factor β
TLR	Toll-like receptor
TNF	tumor necrosis factor
TNFRSF12A	TNF receptor superfamily member 12A
TPM	transcripts per million
Tregs	regulatory T cells
TXNIP	thioredoxin-interacting protein
VEGF	vascular endothelial growth factor
